# Performance Guarantees of Recurrent Neural Networks for the Subset Sum Problem

**DOI:** 10.3390/biomimetics10040231

**Published:** 2025-04-08

**Authors:** Zengkai Wang, Weizhi Liao, Youzhen Jin, Zijia Wang

**Affiliations:** 1 College of Artificial Intelligence, Jiaxing University, Jiaxing 314001, China; wangzengkai@zjxu.edu.cn (Z.W.); jinritian521@sina.com (Y.J.); 2 School of Computer Science and Cyber Engineering, Guangzhou University, Guangzhou 510006, China

**Keywords:** subset sum problem, performance guarantee, deep learning, recurrent neural networks, dynamic programming

## Abstract

The subset sum problem is a classical NP-hard problem. Various methods have been developed to address this issue, including backtracking techniques, dynamic programming approaches, branch-and-bound strategies, and Monte Carlo methods. In recent years, researchers have proposed several neural network-based methods for solving combinatorial optimization problems, which have shown commendable performance. However, there has been limited research on the performance guarantees of recurrent neural networks (RNNs) when applied to the subset sum problem. In this paper, we conduct a novel investigation into the performance guarantees of RNNs to solve the subset sum problem for the first time. A construction method for RNNs is developed to compute both exact and approximate solutions of subset sum problems, and the mathematical model of each hidden layer in RNNs is rigorously defined. Furthermore, the correctness of the proposed RNNs is strictly proven through mathematical reasoning, and their performance is thoroughly analyzed. In particular, we prove $w^{NN}\geq w^{OPT}\left(1-\varepsilon \right)$ mathematically, i.e., the errors between the approximate solutions obtained by the proposed ASS-NN model and the actual optimal solutions are relatively small and highly consistent with theoretical expectations. Finally, the validity of RNNs is verified through a series of examples, where the actual error value of the approximate solution aligns closely with the theoretical error value. Additionally, our research reveals that recurrence relations in dynamic programming can effectively simulate the process of constructing solutions.

## 1. Introduction

The subset sum problem (SSP) is a well-established issue in combinatorial optimization problems (COPs), which has found extensive applications in various engineering domains, including capital budgeting, workload allocation, and job scheduling. While the solution to the verification aspect of this problem is relatively straightforward, identifying a subset of numbers that satisfies a specified target remains a challenging endeavor.

### 1.1. Heuristic Algorithm for SSP

Over the past few decades, numerous exact and heuristic algorithms have been developed to address the SSP [1,2]. M. F. M. K. Madugula et al. proposed a well-enabled meta-heuristic algorithm named the arithmetic optimization algorithm to solve the SSP [3], while Wan et al. proposed an efficient GPU-based parallel double-list algorithm to solve the SSP, achieving higher performance gains on GPUs than CPUs by improving the design of generation, pruning, and search phases and optimizing GPU memory management and task allocation [4,5]. P. Dutta et al. presented search algorithms for variants of the SSP [6], and Ye et al. introduced a priority algorithm approximation ratios for the SSP with a focus on the power of revocable decisions, where accepted data items can be later rejected to maintain solution feasibility [7]. V. Parque developed a new scheme to sample solutions for the SSP based on swarm-based optimization algorithms with distinct forms of selection pressure, the balance of exploration-exploitation, the multi-modality considerations, and a search space defined by numbers associated with subsets of fixed size [8]. L. Li et al. devised a DNA procedure for solving the SSP in the Adleman–Lipton model, which operates in O(n) steps for an undirected graph with n vertices [9]. J. R. M. Kolpakov et al. presented a readily implementable recursive parallelization strategy for solving the SSP using the branch-and-bound method [10]. R. Kolpakov et al. studied the question of parallelization of a variant of the branch-and-bound method for solving the SSP [11]. Thada et al. proposed a genetic algorithm approach with infeasible offspring rejection and a penalty function to find approximate solutions for the SSP, demonstrating improved efficiency by discarding unfit subsets during the optimization process [12]. Li et al. presented a DNA-based algorithm in the Adleman–Lipton model that solves the SSP in O(n) time by strategically designing vertex strand lengths to simplify computation and efficiently identify valid subsets [9]. Wang et al. proposed an enhanced genetic algorithm for the SSP that replaces probabilistic operations with conditional crossover and mutation, demonstrating improved capability to find optimal solutions [13]. Bhasin et al. proposed a genetic algorithm-based approach to solve the SSP and explored its potential as a generic methodology for tackling NP-complete problems, demonstrating promising results through implementation and analysis [14]. Saketh et al. evaluated the genetic algorithm for solving the SSP and compared it with dynamic programming, concluding that the genetic algorithm is less favorable due to its longer execution time despite its adaptability [15]. Genetic algorithms exhibit strong adaptability when addressing large-scale problems. However, their performance is sensitive to parameter selection, and they are susceptible to becoming trapped in local optima.

### 1.2. Dynamic Programming for SSP

Dynamic programming is an effective method for solving COPs [16]. The basic idea of dynamic programming is to decompose the problem into several sub-problems with similar structures and then derive the solution of the original problem from the solutions of these sub-problems. Yang et al. proposed a neural network-based dynamic programming method for NP-hard problems. Since this method requires training on each testing instance of the problem, its time complexity remains high for practical tasks [17]. Allock et al. put forward a novel dynamic programming data structure with applications to subset-sum and its variants including equal-sums, two-subset-sum, and shifted-sums [18], while H. Fujiwara et al. formalized the recurrence relation of the dynamic programming for the SSP [19]. Xu et al. developed a general framework called neural network approximated dynamic programming, which replaces policy or value function calculation processes with neural networks [20]. Both Yang et al.’s and Xu et al.’s studies utilized neural networks to expedite dynamic programming algorithms for COPs but not to search solution space. This proposed method can be generalized to other COPs that can be solved by dynamic programming. However, different problems have different dynamic programming equations, and different COPs have different approximate solution methods. Therefore, correctly establishing corresponding neural networks for specified COPs is crucial in this approach. The dynamic programming method constructs solutions based on recursive relationships, enabling the effective acquisition of optimal solutions. However, even when problems are broken down into sub-problems to reduce search space, the complexity of algorithms using dynamic programming can still be high for NP-hard problems [20]. However, its time and space complexity increases significantly with the growth of the problem size, making it more suitable for solving small-scale problems.

### 1.3. Others

Biesner et al. tackled the SSP as a quadratic unconstrained binary optimization problem and showed how gradient descent on Hopfield networks reliably determines solutions for both artificial and real data [21]. Chenyang Xu and Guochuan Zhang introduced an enhanced learning algorithm to address the online SSP. This approach is designed for rapid solution generation, which does not guarantee the accuracy of the solutions obtained [22]. Coron J. et al. described a proven polynomial-time algorithm for solving the hidden SSP based on statistical learning [23]. M. Costandin provided a geometric interpretation of a specific class of SSP [24]. Zheng Q. L. et al. proposed a method for solving the SSP utilizing a quantum algorithm. However, this approach is only effective for collections containing a limited number of elements [25], and in [26], they translated SSP into the quantum Ising model and solved it with a variational quantum optimization method based on conditional values at risk. This method provides a new perspective for solving the SSP. Moon explored the potential of quantum computing, particularly Grover’s algorithm, to solve the NP-complete SSP more efficiently than classical methods like dynamic programming while also discussing the broader implications for NP-complete problems [27]. Bernstein et al. introduced a new algorithm that combines the Howgrave-Graham–Joux subset sum algorithm with a new streamlined data structure for quantum walks on Johnson graphs [28]. Quantum algorithms leverage superposition states to simultaneously explore multiple candidate solutions, allowing for efficient searches of optimal solutions within the solution space. Nevertheless, this approach is prone to noise interference, has high implementation complexity, and requires specialized quantum equipment.

With the advancement in deep learning and neural networks, significant progress has been made in the application of deep learning to fields such as image recognition, natural language processing, and autonomous driving. As a result, there is growing interest in exploring the potential of deep learning in other domains. Research indicates that deep learning and neural networks show promise in addressing COPs [29]. Notable efforts to apply deep learning methodologies to COPs are summarized in [30]. Recurrent neural networks (RNNs) are powerful sequence models. Hopfiled et al. were among the first to utilize RNNs to solve COPs [31]. M. S.Tarkov proposed an algorithm for solving the traveling salesman problem based on the use of the Hopfield recurrent neural network, the “Winner takes all” method for the cycle formation, and the two-opt optimization method [32]. Gu et al. devised a novel algorithm for the L smallest k-subsets sum problem by integrating the L shortest paths algorithm with the finite-time convergent recurrent neural network [33]. Gu and Hao applied recurrent neural networks driven by pure data to solve the 0–1 knapsack problems [34]. Zhao et al. proposed a two-phase neural combinatorial optimization method with reinforcement learning for the agile Earth-observing satellite scheduling problem [35]. M. T. Kechadi et al. developed an efficient neural network approach to minimize the cycle time for job shop scheduling problems [36]. C. Hertrich et al. conducted a study on the expressive power of neural networks through knapsack problems. They iteratively applied a class of RNNs to each item in a knapsack instance and computed optimal or provably good solution values [37].

Neural networks demonstrate significant advantages in solving COPs, such as robust pattern-learning capabilities and the ability to uncover hidden rules. By employing end-to-end mapping, neural networks bypass the explicit traversal of all possible composition spaces, thereby avoiding the issue of combinatorial explosion. These characteristics are absent in traditional heuristic algorithms and dynamic programming methods. Nonetheless, neural networks also possess certain limitations, including high dependence on data, substantial costs associated with generating high-quality training samples, and excessive computational resources required for exact solutions in some cases. Additionally, the accuracy of approximate solutions remains an area requiring improvement.

### 1.4. Our Contributions

In this paper, we present a rigorous mathematical study on the construction of RNNs to solve two types of SSPs. The main contributions of this paper are as follows:

1. We introduce a recurrent neural network, denoted as SS-NN, to solve the classical SSP. By defining a new activation function, we develop a dynamic programming equation for the classical SSP. This activation function maps all negative numbers to −1 and ensures that the number of inputs to the neural network is fixed. NN-SS is utilized to mimic the dynamic programming approach for solving the SSP. We define the mathematical model for each hidden layer of NN-SS and prove its correctness.
2. We propose an approximate solution method for a type of SSP, where the value of the subset-sum is closest to a given value but not exceeding it. The dynamic programming equations for this type of SSP are defined using rounding granularity and the ReLU activation function. We construct a recurrent neural network, denoted as ASS-NN, to mimic the presented dynamic programming approach in determining an approximate solution. We rigorously prove that our proposed ASS-NN can correctly solve the SSP and analyze both its time complexity and error in approximation.
3. We verify the correctness of our proposed method through examples and demonstrate that actual error values in approximate solutions align with theoretical error values through a series of illustrative examples.

The remainder of this paper is structured as follows. Section 2 describes the construction of a recurrent neural network to solve the classical SSP. Section 3 presents an approximate solution method for another type of SSP by constructing a novel recurrent neural network. Section 4 provides the experimental results and performance error analysis. Section 5 discusses the work of this paper in depth. Finally, we conclude this paper and discuss future work in Section 6.

## 2. RNNs for an Exact Solution to the SSP

In this section, we aim to determine whether a given set of positive numbers *S* contains a subset whose sum equals a specified positive number *W*. This problem can be formulated as a YES/NO decision problem, denoted as the Y/N SSP in the following discussion. It is worth noting that this problem falls within the class of NP-complete problems [38]. Let $S=\{w_{1},\ldots ,w_{n}\}$ be a subset of $N^{+}$ and $W\in N^{+}$ be a fixed number. Let $S^{*}=\left\{w_{j_{1}},\ldots ,w_{j_{m}}\right\}$ be a subset of *S*, and $\;V^{*}=\sum_{k=j_{1}}^{j_{m}}w_{k}$. The mathematical model of the Y/N SSP is described by Equation (1).

(1) $$y=\left\{\begin{array}{cc} 1 & \exists S^{*}\subseteq S\;\text{satisfies}\;V^{*}=W\\ 0 & otherwise \end{array}.\right.$$

We propose a dynamic programming formulation for the Y/N SSP. Let $s\left(w,i\right)\in \left\{0,1\right\}$. If the sum of the first *i* elements of set *S* equals *w*, then s(w,i)=1; otherwise, s(w,i)=0. Notably, s(w,i)=1 when w=0. The values of s(w,i) can be computed using Equation (2).

(2) $$s\left(w,i\right)=\left\{\begin{array}{cc} s(w,i-1) & w<w_{i}\\ s\left(w-w_{i},i-1\right)\;\;or\;s\left(w,i-1\right) & w\geq w_{i} \end{array},\right.$$

where $i\in \left[n\right]$, and $\left[n\right]=\left\{1,2,\ldots ,n\right\}$. If s(w,i) = 1, the corresponding subset can be obtained by backtracking.

Let $s(w-w_{i},i-1)=0$ for $w-w_{i}<0$. It is evident that Equation (2) can be substituted with Equation (3) as follows:

(3) $$s\left(w,i\right)=s\left(w-w_{i},i-1\right)\;\;or\;s\left(w,i-1\right).$$

Notably, s(w,i)=1 for w=0.

Inspired by the work of [37], who employed a recurrent neural network to address the knapsack problem, we present our own recurrent neural network, referred to as SS-NN, aimed at solving the Y/N SSP in this section. It is evident that if $w-w_{i}<0$, then $s(w-w_{i},i-1)=0$. However, it is impractical to input all possible values of $w-w_{i}$ into the neural network. Therefore, we represent all negative numbers with −1. Consequently, we define a novel activation function $\delta^{\prime }\left(x\right)$, as shown in Equation (4).

(4) $$\delta^{\prime }\left(x\right)=\left\{\begin{array}{cc} x & x\geq 0\\ -1 & x<0 \end{array}.\right.$$

**Figure 1:**
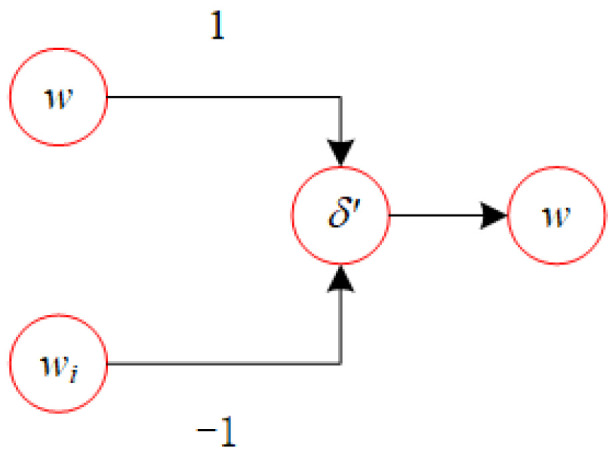
Network for the $\delta^{\prime }$ function.

According to the activation function $\delta^{\prime }\left(x\right)$, if $w-w_{i}\geq 0$, then we have $\delta^{\prime }\left(w-w_{i}\right)=\;w-w_{i}$. Conversely, if $w-w_{i}<0$, then it follows that $\delta^{\prime }\left(w-w_{i}\right)=-1$. The network structure for $\delta^{\prime }\left(x\right)$ is shown in Figure 1.

Therefore, we can utilize Equation (5) to replace Equation (3) with the activation function $\delta^{\prime }\left(x\right)$. The definition of Equation (5) is as follows:

(5) $$s\left(w,i\right)=s\left(\delta^{\prime }\left(w-w_{i}\right),i-1\right)\;\;or\;s\left(w,i-1\right).$$

Notably, s(−1,0)=0, s(0,0)=0, and $s(w^{*},0)=0$ for $w^{*}>0$.

**Figure 2:**
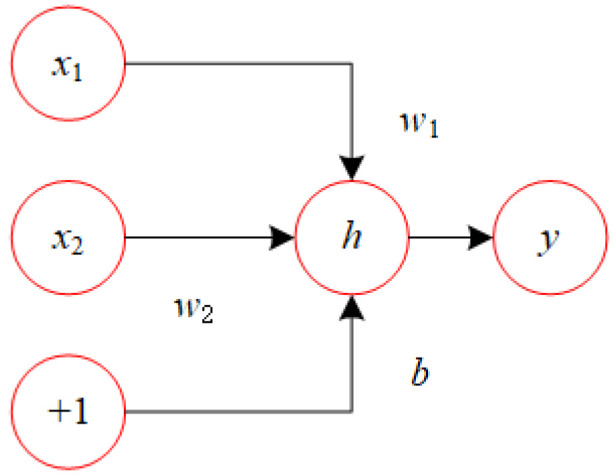
Network for or operator.

In order to solve the Y/N SSP by implementing the logical disjunction operation, we have developed a neural network specifically designed for this purpose. The network structure for $x_{1}$ or $x_{2}$ is illustrated in Figure 2, with the activation function h(x) as a step function. The precise definition of h(x) can be found in Equation (6).

(6) $$h\left(x\right)=\left\{\begin{array}{cc} 1 & x>0\\ 0 & x\leq 0 \end{array}.\right.$$

**Figure 3:**
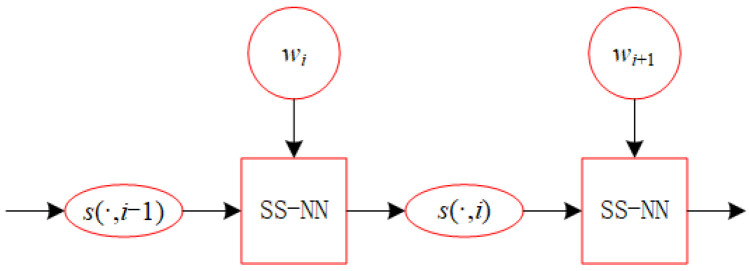
Recurrent structure based on SS-NN.

**Figure 4:**
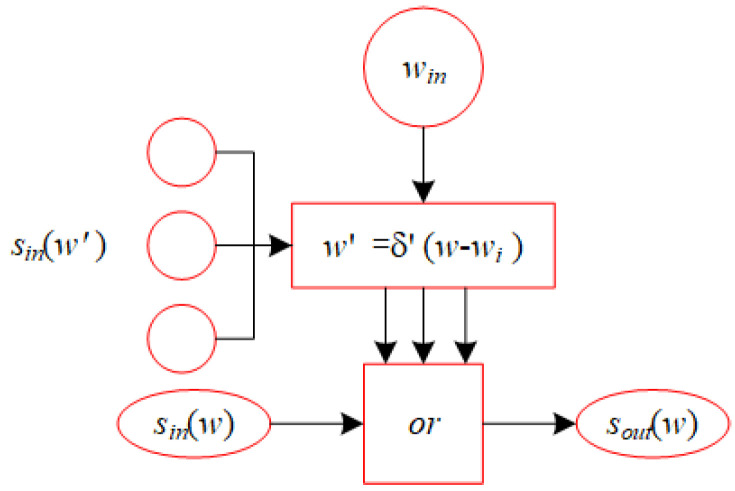
The structure of SS-NN.

The recurrent structure based on SS-NN is shown in Figure 3. An overview of the entire structure of SS-NN can be seen in Figure 4.

To clearly demonstrate the recurring structure of SS-NN, we will omit the use of the index *i* in the following description of the recurrent neural network. Equation (5) will be replaced by Equation (7).

(7) $$s_{out}\left(w\right)=s_{in}\left(\delta^{\prime }\left(w-w_{in}\right)\right)\;or\;s_{in}\left(w\right).\;$$

The inputs of SS-NN consist of $s_{in}\left(-1\right),s_{in}\left(0\right),s_{in}\left(1\right),\ldots ,s_{in}\left(W\right)$, and $w_{in}$. Therefore, the input layer has W+3 neurons, where the values of $s_{in}\left(-1\right)=0$ and $s_{in}\left(0\right)=1$ are constants.

### 2.1. The First Hidden Layer of SS-NN

The first hidden layer is utilized for computing $\delta^{\prime }\left(w-w_{in}\;\right)$ and consists of W+2 neurons, denoted as $F_{1}\left(w,w_{in}\right)$ for $w\in \left\{-1,0\right\}\cup \left[W\right]$, where $w_{in}\in N^{+}$, and $\left[W\right]=\left\{1,2,\ldots ,W\right\}$, $N^{+}$ represents the set of positive integer. The neurons are defined as follows:

(8) $$F_{1}\left(w,w_{in}\right)=\delta^{\prime }\left(w-w_{in}\;\right),w\in \left\{-1,0\right\}\cup \left[W\right].$$

**Theorem** **1.** *If $w\in \left\{0,1\right\}\cup \left[W\right]$, and $w_{in}\in N^{+}$, then $F_{1}\left(w,w_{in}\right)$ belongs to the set {−1,0,1,⋯,w−1}.*

**Proof.**  We observe that $F_{1}\left(w,w_{in}\right)=\delta^{\prime }\left(w-w_{in}\;\right)$. Thus, if $w-w_{in}<0$, we have $F_{1}\left(w,w_{in}\right)=-1$ according to the definition $\delta^{\prime }$ function. If $w-w_{in}\geq 0$, then $F_{1}\left(w,w_{in}\right)=w-w_{in}$. Since $w_{in}\in N^{+}$, it is evident that the maximum value of $w-w_{in}$ is equal to *w* − 1. Therefore, we can conclude that the value $F_{1}\left(w,w_{in}\right)$ must belong to −1,0,1,⋯,w−1. □

### 2.2. The Second Hidden Layer of SS-NN

The second hidden layer contains $2\left(W+2\right)*W+2$ neurons, denoted as $F_{2}^{\left(+\right)}\left(w,w^{\prime }\right)$ and $F_{2}^{\left(-\right)}\left(w,w^{\prime }\right)$, where $w^{\prime }$ and *w* belong to the set $\left\{-1,0\right\}\cup \left[W\right]$.

(9) $$F_{2}^{\left(+\right)}\left(w,w^{\prime }\right)=\delta \left(2\left(F_{1}\left(w,w_{in}\right)-w^{\prime }\right)\right),$$

(10) $$F_{2}^{\left(-\right)}\left(w,w^{\prime }\right)=\delta \left(2\left(w^{\prime }-F_{1}\left(w,w_{in}\right)\right)\right),\;$$

where δ(x) represents the ReLu activation function. The objective of the second hidden layer is to ascertain whether the value $w^{\prime }$ is equal to $F_{1}\left(w,w_{in}\right)$ or not.

**Theorem** **2.** *$\;F_{2}^{\left(+\right)}\left({w,w}^{\prime }\right)$ + $F_{2}^{\left(-\right)}\left(w,w^{\prime }\right)=0$ if only if $w^{\prime }=F_{1}\left(w,w_{in}\right)$. Otherwise, we have $\;F_{2}^{\left(+\right)}\left({w,w}^{\prime }\right)$ + $F_{2}^{\left(-\right)}\left({w,w}^{\prime }\right)\geq 2$.*

**Proof.**  Clearly, if $w^{\prime }=F_{1}\left(w,w_{in}\right)$, then we have $F_{1}\left(w,w_{in}\right)-w^{\prime }=0$ and $w^{\prime }-F_{1}\left(w,w_{in}\right)$ = 0. Consequently, we derive that $F_{2}^{\left(+\right)}\left(w,w^{\prime }\right)$ = 0 and $\;F_{2}^{\left(-\right)}\left({w,w}^{\prime }\right)$ = 0. Therefore, it follows that $F_{2}^{\left(+\right)}\left({w,w}^{\prime }\right)$ + $F_{2}^{\left(-\right)}\left({w,w}^{\prime }\right)$ = 0.

If $F_{2}^{\left(+\right)}\left(w,w^{\prime }\right)$ + $F_{2}^{\left(-\right)}\left({w,w}^{\prime }\right)$ = 0, and given that $F_{2}^{\left(+\right)}\left({w,w}^{\prime }\right)\geq 0$ and $F_{2}^{\left(-\right)}\left({w,w}^{\prime }\right)\geq 0$, we can conclude that $F_{2}^{\left(+\right)}\left({w,w}^{\prime }\right)=0\;,F_{2}^{\left(-\right)}\left(w,w^{\prime }\right)=0$. Therefore, we have established that $\;w^{\prime }=F_{1}\left(w,w_{in}\right)$.

If $w^{\prime }\neq F_{1}\left(w,w_{in}\right)$, then we have either $w^{\prime }>F_{1}\left(w,w_{in}\right)$ or $w^{\prime }<F_{1}\left(w,w_{in}\right)$. In the case where $w^{\prime }>F_{1}\left(w,w_{in}\right)$, it follows that $w^{\prime }-F_{1}\left(w,w_{in}\right)\geq 1$ since $w^{\prime }\in \left\{1,0\right\}\cup \left[W\right]$ and $\;F_{1}\left(w,w_{in}\right)\in \left\{-1,0,1,\cdots ,w-1\right\}$. Consequently, we determine that $F_{2}^{\left(+\right)}\left({w,w}^{\prime }\right)=0$ and $F_{2}^{\left(-\right)}\left({w,w}^{\prime }\right)\geq 2$. Thus, it holds that $\;F_{2}^{\left(+\right)}\left({w,w}^{\prime }\right)$ + $F_{2}^{\left(-\right)}\left({w,w}^{\prime }\right)\geq 2$. Conversely, if $w^{\prime }<F_{1}\left(w,w_{in}\right)$, we also conclude that $F_{2}^{\left(+\right)}\left(w^{\prime }\right)$ + $F_{2}^{\left(-\right)}\left(w^{\prime }\right)\geq 2$.

Let $F_{2}^{\left(+\right)}\left(w^{\prime }\right)$ + $F_{2}^{\left(-\right)}\left(w^{\prime }\right)\geq 2$. We assume that $w^{\prime }=F_{1}\left(w,w_{in}\right)$. Consequently, we have $F_{2}^{\left(+\right)}\left(w,w^{\prime }\right)$ + $F_{2}^{\left(-\right)}\left({w,w}^{\prime }\right)=0$, which contradicts the inequality $F_{2}^{\left(+\right)}\left({w,w}^{\prime }\right)$ + $F_{2}^{\left(-\right)}\left({w,w}^{\prime }\right)\geq 2$. Thus, our initial hypothesis is invalid and we conclude that $w^{\prime }\neq F_{1}\left(w,w_{in}\right)$. □

### 2.3. The Third Hidden Layer of SS-NN

The third hidden layer contains (W+2)∗W+2 neurons, denoted as $F_{3}\left(w,k\right)$ for *w* and *k*$\in \left\{-1,0\right\}\cup \left[W\right]$. These neurons are defined as follows:

(11) $$F_{3}\left(w,k\right)=\delta \left(s_{in}\left(k\right)-F_{2}^{\left(+\right)}\left(w,k\right)-F_{2}^{\left(-\right)}\left(w,k\right)\right).$$

**Theorem** **3.** *For $w,k\in \left\{-1,0\right\}\cup \left[W\right]$ and $w_{in}\in N^{+}$, we have $F_{3}\left(w,k\right)=s_{in}\left(k\right)$ if only if $k=F_{1}\left(w,w_{in}\right)$. Otherwise, it follows that $F_{3}\left(w,k\right)=0$.*

**Proof.**  If $k=F_{1}\left(w,w_{in}\right)$, then by Theorem 2, we have $F_{2}^{\left(+\right)}\left(w,k\right)$ + $F_{2}^{\left(-\right)}\left(w,k\right)$ = 0. This leads to the conclusion that $s_{in}\left(k\right)-F_{2}^{\left(+\right)}\left(w,k\right)-F_{2}^{\left(-\right)}\left(w,k\right)=s_{in}\left(k\right)$. Therefore, if $k=F_{1}\left(w,w_{in}\right)$, it follows that $F_{3}\left(w,k\right)=s_{in}\left(k\right)$ by $s_{in}\left(k\right)\in \left\{0,1\right\}$.

Conversely, if $k\neq F_{1}\left(w,w_{in}\right)$, we obtain from Theorem 2 that $F_{2}^{\left(+\right)}\left(w,k\right)$ + $F_{2}^{\left(-\right)}\left(w,k\right)\geq 2$. Since $s_{in}\left(k\right)\in \left\{0,1\right\}$, this implies that $\;s_{in}\left(k\right)-F_{2}^{\left(+\right)}\left(w,k\right)-F_{2}^{\left(-\right)}\left(w,k\right)<0$. Thus, we conclude that $\;F_{3}\left(w,k\right)=0$. □

### 2.4. The Fourth Hidden Layer of SS-NN

The fourth hidden layer contains W+2 neurons, denoted as $F_{4}\left(w\right)$ for w and k $\in \left\{-1,0\right\}\cup \left[W\right]$.

(12) $$F_{4}\left(w\right)=\sigma \left(\sum_{k=-1}^{W}F_{3}\left(w,k\right)\right).$$

**Theorem** **4.** *For each $w,k\in \left\{-1,0\right\}\cup \left[W\right]$ and $w_{in}\in N^{+}$, we have $s_{in}\left(\delta^{\prime }\left(w-w_{in}\;\right)\right)=F_{4}\left(w\right)=\sigma \left(\sum_{k=-1}^{W}F_{3}\left(w,k\right)\right)$.*

**Proof.**  According to the definition of $\delta^{\prime }\left(x\right)$, we have

(13) $$s_{in}\left(\delta^{\prime }\left(w-w_{in}\;\right)\right)=\left\{\begin{array}{cc} 0\;\;\;\;\;\;\;\;\;\;\;\;\;\;\;\;\;\;\;\;\;\; & w-w_{in}<0\\ s_{in}\left(w-w_{in}\right) & w-w_{in}>0\\ 1\;\;\;\;\;\;\;\;\;\;\;\;\;\;\;\;\;\;\;\;\; & w-w_{in}=0 \end{array}.\right.$$

On the other hand, if $k>F_{1}\left(w,w_{in}\right)$ or $k<F_{1}\left(w,w_{in}\right)$, we have $F_{2}^{\left(+\right)}\left(w,k\right)$ + $F_{2}^{\left(-\right)}\left(w,k\right)\geq 2$. Since $s_{in}\left(k\right)\in \left\{-1,0\right\}$, this implies that $\;F_{3}\left(w,k\right)=0$. Consequently, we determine

$\sum_{k=-1\;and\;k\neq F_{1}\left(w,w_{in}\right)}^{W}F_{3}\left(w,k\right)=0$. Thus, the value $\sum_{k=-1}^{W}F_{3}\left(w,k\right)$ is determined by *k*, where $\;k=F_{1}\left(w,w_{in}\right)$. We can draw the following conclusions.

1. If $k=F_{1}\left(w,w_{in}\right)=0$, then it follows that $w-w_{in}=0$ and $F_{3}\left(w,k\right)=\;\delta \left(s_{in}\left(k\right)\right)=\delta \left(s_{in}\left(0\right)\right)$ = 1. Hence, we obtain $F_{4}\left(w\right)=\sigma \left(\sum_{k=-1}^{W}F_{3}\left(w,k\right)\right)=\delta \left(1\right)=1$.
2. If $k=F_{1}\left(w,w_{in}\right)=-1$, then we conclude that $w-w_{in}<0\;\mathrm{and}\;F_{3}\left(w,k\right)=\;\delta \left(s_{in}\left(k\right)\right)=\delta \left(s_{in}\left(-1\right)\right)$ = 0. Therefore, we have $F_{4}\left(w\right)=\sigma \left(\sum_{k=-1}^{W}F_{3}\left(w,k\right)\right)=\delta \left(0\right)=0$.
3. If $k=F_{1}\left(w,w_{in}\right)>0$, this implies that $k=w-w_{in}>0$ and we have $F_{3}\left(w,k\right)=\;\delta \left(s_{in}\left(k\right)\right)=\delta \left(s_{in}\left(w-w_{in}\right)\right)=s_{in}\left(w-w_{in}\right)$. Thus, we conclude that $F_{4}\left(w\right)=\sigma \left(\sum_{k=-1}^{W}F_{3}\left(w,k\right)\right)=\delta \left(s_{in}\left(w-w_{in}\right)\right)=s_{in}\left(w-w_{in}\right)$.

In summary, it holds true that $F_{4}\left(w\right)=s_{in}\left(\delta^{\prime }\left(w-w_{in}\right)\right)$. This indicates that the fourth hidden layer is capable of accurately computing the value of $s_{in}\left(\delta^{\prime }\left(w-w_{in}\right)\right)$. □

### 2.5. The Fifth Hidden Layer of SS-NN

The fifth hidden layer is used to compute $s_{in}\left(\delta^{\prime }\left(w-w_{in}\right)\right)\;or\;s_{in}\left(w\right)$, and it contains W+2 neurons, denoted as $F_{5}\left(w\right)$ for $w\in \left\{-1,0\right\}\cup \left[W\right]$. The definitions are as follow:

(14) $$F_{5}\left(w\right)=s_{in}\left(\delta^{\prime }\left(w-w_{in}\right)\right)\;or\;s_{in}\left(w\right),$$

(15) $$s_{out}\left(w\right)=F_{5}\left(w\right).$$

**Theorem** **5.** *For each w∈{−1,0}∪([W] and $w_{\mathrm{in}}\in N^{+}$, if $w<w_{in}$, then we have $F_{5}\left(w\right)=s_{in}\left(w\right)$. Conversely, if $w\geq w_{in}$, it follows that $F_{5}\left(w\right)=s_{in}\left(w-w_{in}\right)$ or $s_{in}\left(w\right)$.*

**Proof.**  If $w<w_{in}$, i.e., $w-w_{in}<0$, then we have $\delta^{\prime }\left(w-w_{in}\right)=-1$. Thus, we obtain $s_{in}\left(\delta^{\prime }\left(w-w_{in}\right)\right)=s_{in}\left(-1\right)=0$. Consequently, it follows that $F_{5}\left(w\right)=s_{in}\left(\delta^{\prime }\left(w-w_{in}\right)\right)$ or $s_{in}\left(w\right)=0$ or $s_{in}\left(w\right)=s_{in}\left(w\right)$.

Conversely, if $w\geq w_{in}$, i.e., $w-w_{in}\geq 0$, then we determine that $\delta^{\prime }\left(w-w_{in}\right)=w-w_{in}$, leading to $s_{in}\left(\delta^{\prime }\left(w-w_{in}\right)\right)=s_{in}\left(w-w_{in}\right)$. Therefore, we conclude that $F_{5}\left(w\right)=s_{in}\left(w-w_{in}\right)$ or $s_{in}\left(w\right)$. □

**Theorem** **6.** *Let $S=\{w_{1},\ldots ,w_{n}\}$, where $w_{i}\in N^{+}\left(1\leq i\leq n\right)$, and let W be a positive integer. We can utilize the SS-NN to iteratively compute the value of s(w,i) for w∈{−1,0}∪[W], i∈[n].*

**Proof.**  We observe that s(−1,0)=0, s(0,0)=1, and $s(w_{1},0)=0$ for $w_{1}>0$. In the first iteration, $w_{in}=w_{1}$. Since $0<w_{in}$, it follows that s(−1,1)=s(−1,0)=0, and s(0,1)=s(0,0)=1 according to Theorem 4. For w∈[W], if $w<w_{in}$, we have s(w,1)=s(w,0), otherwise we obtain $s\left(w,1\right)=s\left(w-w_{in},0\right)$ or $s(w_{in},0)$ by Theorem 4. In the second iteration, since $w_{in}=w_{2}$, we have s(−1,2)=s(−1,1)=0, and s(0,2)=s(0,1)=1 by Theorem 4. For any w∈[W], if $w<w_{in}$, it follows that s(w,2)=s(w,1). Conversely, if this condition does not hold true, we conclude that $s\left(w,2\right)=s\left(w-w_{in},1\right)$ or s(w,1) and so on. In the final iteration, where $w_{in}=w_{n}$, we have s(−1,n)=s(−1,n−1)=0, and s(0,n)=s(0,n−1)=1 by Theorem 4. For any w∈[W], if $w<w_{in}$, we have s(w,n)=s(w,n−1); otherwise, we have $s\left(w,n\right)=s\left(w-w_{in},n-1\right)$ or s(w,n−1). □

The depth of a single SS-NN is seven, comprising five hidden layers, one input layer, and one output layer. Consequently, the overall depth of the SS-NN designed for solving SSPs is 7n, where *n* represents the number of elements in the set. Thus, the order of depth can be expressed as O(7n). The width of the SS-NN is determined by the value of *W*. Given that the hidden layers with maximum width are the second and third layers, we determine that the width order for a single SS-NN is $O(2{\left(W+2\right)}^{2})$. Therefore, when unfolding the SS-NN and treating it as a singular neural network executing an entire dynamic program, we arrive at a width complexity of $O(2n{\left(W+2\right)}^{2})$.

## 3. RNNs for the Approximate Solution to the SSP

It is not hard to know that the exact result of an SSP is dependent on the network width *M*. In order to overcome this drawback, we proposed a neural network called ASS-NN that can determine the approximation solution for variants of SSP. Similar to [37], ASS-NN uses less neurons but loses optimality. Let $S=\{w_{1},\ldots ,w_{n}\}$ be a subset of $N^{+}$, $P\in N^{+}$ be a fixed number, $S^{*}=\left\{w_{j_{1}},\ldots ,w_{j_{m}}\right\}$ be a subset of *S*, and $V^{*}=\sum_{k=j_{1}}^{j_{m}}w_{k}$. In this section, our task is determining subset $S^{*}$ such that $V^{*}$ is closest to *P* but not more than *P*. This kind of SSP is more common in practical applications. Let $w_{i}^{*}=\sum_{k=1}^{i}w_{k}$ be the total value of the first *i* elements. Once $w_{i}^{*}$ exceed *P*, we do not store a required value for every possible sum but have to round sum. The rounding granularity of $w_{i}^{*}$ is denoted by $r_{i}=\mathrm{roundup}\left(\max\left\{1,\frac{w_{i}^{*}}{M}\right\}\right)$, where roundup(x) is the upward rounding function and $M\in N^{+}$. Let p(w,i) be the total value of a subset of *S*, and it must be closest to $wr_{i}$ but not more than $wr_{i}$, where $w\in \left[M\right]$. The values of p(w,i) can be computed recursively by Equation (16).

(16) $$p\left(w,i\right)=\left\{\begin{array}{c} \max\left\{p^{\left(1\right)},p^{\left(2\right)}\right\},\exists w^{\left(2\right)}\in M\;\mathrm{such}\;\mathrm{that}\;p^{\left(2\right)}\leq wr_{i}\\ p^{\left(1\right)},\mathrm{otherwise} \end{array}\right.,$$

where $p^{\left(1\right)}=\;p\left(w^{\left(1\right)},i-1\right)$ and $p^{\left(2\right)}=p\left(w^{\left(2\right)},i-1\right)+w_{i}$. Let ${w^{\left(1\right)},w}^{\left(2\right)}\geq 1,w^{\left(1\right)}$ be the largest possible integers satisfying the condition $p\left(w^{\left(1\right)},i-1\right)\leq wr_{i}$, and $w^{\left(2\right)}\in M\;$ be the largest possible integers that satisfies the condition $p\left(w^{\left(2\right)},i-1\right)+w_{i}\leq wr_{i}$. In particular, if i≤0, then $p\left(w,i\right)=0$. In order to use a recurrent neural network to determine the solution for Equation (16), we introduce an activation function called ReLUs to reconstruct dynamic programming for the SSP. The solution can be computed recursively by Equation (17).

(17) $$\begin{array}{cc} \; & p\left(w,i\right)=\max\left\{p\left(w^{\left(1\right)},i-1\right),\delta \left(p\left(w^{\left(2\right)},i-1\right)+w_{i}\right)\right\},\; \end{array}$$

where $p\left(w,\;\;i\right)=-\infty$ for w≤0 and $p\left(w,0\right)=0$, $w^{\left(1\right)}$ is the largest possible integer satisfying the condition $p\left(w^{\left(1\right)},i-1\right)\leq wr_{i}$, and $w^{\left(2\right)}$ is the largest possible integer that satisfies the condition $p\left(w^{\left(2\right)},i-1\right)$ + $w_{in}\leq wr_{i}$.

**Theorem** **7.** *For w∈[M] and i≥1, the value $p\left(w,i\right)$ in Equation (17) is equal to the value $p\left(w,i\right)$ in Equation (16).*

**Proof.**  Obviously, if w∈[M], then $\;wr_{i}>0$. Since $w^{\left(1\right)}$ is the largest possible integer with $p\left(w^{\left(1\right)},i-1\right)\leq wr_{i}$, it is easy to know that $p\left(w,i\right)\geq 0$ for w∈[M] and i≥1 using Equation (17). Furthermore, we observe that $r_{i-1}\leq r_{i}$. Hence, there exists a value of $\;w^{\left(1\right)}\in \left[M\right]$ satisfying the condition $p\left(0,i-1\right)<p\left(w^{\left(1\right)},i-1\right)\leq w^{\left(1\right)}r_{i-1}\leq wr_{i}$. On the other hand, $w^{\left(1\right)}$ is the largest possible integer satisfying the condition $p\left(\;w^{\left(1\right)},i-1\right)\leq wr_{i}$ according to the definition of Equation (16). Thus, we conclude that the value of $p(w^{\left(1\right)},i-1)$ in Equation (17) is equal to the value of $p(w^{\left(1\right)},i-1)$ in Equation (16).

For Equation (17), if no $w^{\left(2\right)}\in \left[M\right]$ is the largest possible integer satisfying the condition $p\left(w^{\left(2\right)},i-1\right)+w_{in}\leq wr_{i}$, then we have $w^{\left(2\right)}=0$. This leads to $p\left(0,i-1\right)$ + $w_{in}\leq wr_{i}$. Since $p\left(0,i-1\right)=-\infty$, it follows that $\delta (p\left(w^{\left(2\right)},i-1\right)+w_{i})=0$. Consequently, we obtain $p\left(w,i\right)=\max\left\{p\left(w^{\left(1\right)},i-1\right),\delta \left(p\left(w^{\left(2\right)},i-1\right)+w_{i}\right)\;\right\}$ = $p\left(w^{\left(1\right)},i-1\right)$. For Equation (16), if no $w^{\left(2\right)}\in M\;$ is the largest possible integer that satisfies the condition $p\left(w^{\left(2\right)},i-1\right)+w_{i}\leq wr_{i}$, we determine that $\;p\left(w,i\right)=p\left(w^{\left(1\right)},i-1\right)$. Thus, we conclude that the value of $p\left(w,i\right)$ in Equation (17) is equal to the value of $p\left(w,i\right)$ in Equation (16).

For Equation (17), let $\exists w^{\left(2\right)}\in \left[M\right]$ be the largest possible integer that satisfies the condition $p\left(w^{\left(2\right)},i-1\right)+w_{in}\leq wr_{i}$. In this case, we have $\delta \left(p\left(w^{\left(2\right)},i-1\right)+w_{i}\right)=\;p\left(w^{\left(2\right)},i-1\right)+w_{i}$ when $p\left(w^{\left(2\right)},i-1\right)+w_{i}\geq 0$. Consequently, we obtain $\;p\left(w,i\right)=\max\left\{p\left(w^{\left(1\right)},i-1\right),\delta \left(p\left(w^{\left(2\right)},i-1\right)+w_{i}\right)\right)\}=\max\left\{p\left(w^{\left(1\right)}\right),p\left(w^{\left(2\right)},i-1\right)+w_{i}\right\}$. For Equation (16), if $\exists w^{\left(2\right)}\in \left[M\right]$ is the largest possible integer satisfying the condition $p\left(w^{\left(2\right)},i-1\right)+w_{i}\leq wr_{i}$, it follows that $p\left(w,i\right)=\max\left\{p\left(w^{\left(1\right)},i-1\right),\delta \left(p\left(w^{\left(2\right)},i-1\right)+w_{i}\right)\;\right\}$. Thus, we also have the value of $p\left(w,i\right)$ in Equation (17), which is equal to the value of $p\left(w,i\right)$ in Equation (16). □

Furthermore, in order to obviously show the recurrent structure of ASS-NN, we do not use the index *i* in the following:

(18) $$\begin{array}{c} p_{out}\left(w\right)=\max\left\{p_{in}\left(w^{\left(1\right)}\right),\delta \left(p_{in}\left(w^{\left(2\right)}\right)+w_{in}\right)\right\},\;\;w\in M \end{array},$$

where $w_{in}$ represents the value of element *i*, $p_{in}\left(w^{\left(1\right)}\right)$ denotes the option of not using element *i*, and ${\delta (p}_{in}\left(w^{\left(2\right)}\right)+w_{in})$ represents the option of using element *i*. To compute $w^{\left(1\right)}$ and $w^{\left(2\right)}$, both $w_{in}^{*}$ and $w_{in}$ are utilized to determine rounding granularities in ASS-NN.

**Figure 5:**
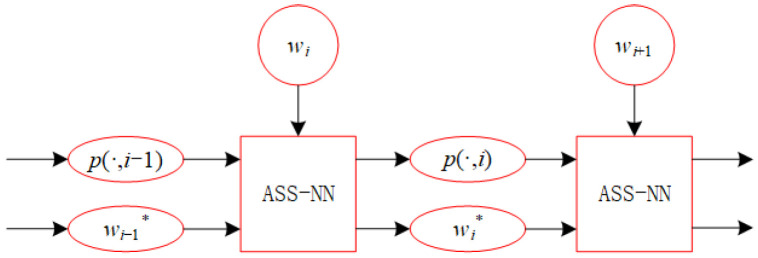
Recurrent structure based on ASS-NN.

**Figure 6:**
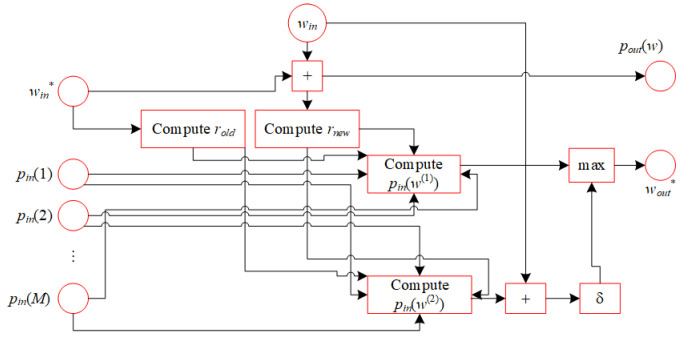
Computing $p_{out}\left(w\right)$ and $w_{out}^{*}$.

In this section, we propose a recurrent neural network referred to as ASS-NN, designed to approximate the solution for Equation (18). The recurrent structure of the proposed neural network is shown in Figure 5. Figure 6 presents the comprehensive structure of ASS-NN, where the first hidden layer is responsible for calculating the previous and current rounding granularities, denoted as $r_{old}$ and $r_{new}$. The second hidden layer’s function is to select integers $w^{\left(1\right)}$ and $w^{\left(2\right)}$. The third hidden layer computes values $p_{in}\left(w^{\left(1\right)}\right)$ and $p_{in}\left(w^{\left(2\right)}\right)$, while the fourth hidden layer determines $\max\{p_{in}\left(w^{\left(1\right)}\right),\delta \left(p_{in}\left(w^{\left(2\right)}\right)+w_{in}\right))\}$.

The ASS-NN is detailed in this section, demonstrating its capability to approximate solutions for the SSP. The proposed neural network comprises four hidden layers.

### 3.1. The First Layer of ASS-NN

The first layer is responsible for calculating the rounding granularities $r_{old}$ and $r_{new}$, which are defined as follows:

(19) $$\begin{array}{c} \begin{array}{c} F_{1}^{\left(1\right)}=\delta \left(\frac{w_{in}^{*}}{M}-1\right),\;F_{1}^{\left(2\right)}=\delta \left(\frac{w_{in}^{*}+w_{in}}{M}-1\right)\\ r_{old}=F_{1}^{\left(1\right)}+1,\;r_{new}=F_{1}^{\left(2\right)}+1 \end{array}. \end{array}$$

The correctness of the computing method was proven in [37].

### 3.2. The Second Layer of ASS-NN

The second layer consists of $2\left(M^{2}+M\right)$ neurons, denoted as $F_{2}^{\left(1+\right)}\left(w,k\right)$ and $F_{2}^{\left(1-\right)}\left(w,k\right)$ for $w\in \left[M\right],k\in \left\{0\right\}\cup \left[M\right]$ with the condition that w≤k. Additionally, we have $F_{2}^{\left(2+\right)}\left(w,k\right)$ and $F_{2}^{\left(2-\right)}\left(w,k\right)$ for $w\in \left[M\right]$, $k\in \left\{0\right\}\cup \left[M\right]$ under the constraint that w≥k.

(20) $$\begin{array}{cc} \; & F_{2}^{\left(1+\right)}\left(w,k\right)=\delta \left(Mr_{max}\left(p_{in}\left(k\right)-wr_{new}\right)\right) \end{array},$$

(21) $$\begin{array}{cc} \; & F_{2}^{\left(1-\right)}\left(w,k\right)=\delta \left(Mr_{max}\left(wr_{new}-p_{in}\left(k+1\right)\right)+r_{max}\right)\; \end{array},$$

(22) $$\begin{array}{cc} \; & F_{2}^{\left(2+\right)}\left(w,k\right)=\delta \left(Mr_{max}\left(p_{in}\left(k\right)+w_{in}-wr_{new}\right)\right)\; \end{array},$$

(23) $$\begin{array}{cc} \; & F_{2}^{\left(2-\right)}\left(w,k\right)=\delta \left(Mr_{max}\left(wr_{new}-p_{in}\left(k+1\right)-w_{in}\right)+r_{max}\right)\; \end{array}.$$

For a fixed $w\in \left[M\right]$, let $w^{\left(1\right)}$ and $w^{\left(2\right)}$ denote the largest possible integers such that $p_{in}\left(w^{\left(1\right)}\right)\leq wr_{new}$ and $p_{in}\left(w^{\left(2\right)}\right)$ + $w_{in}\leq wr_{new}$. The purpose of the second layer is to identify the integers $w^{\left(1\right)}$ and $w^{\left(2\right)}$. The validity of this hidden layer is guaranteed by the following two theorems.

**Theorem** **8.** *For every $w,k\in \left[M\right]$ with w≤k, we have $F_{2}^{\left(1+\right)}\left(w,k\right)$ + $F_{2}^{\left(1-\right)}\left(w,k\right)=0$, if and only if $k=w^{\left(1\right)}$. Otherwise, it follows that $F_{2}^{\left(1+\right)}\left(w,k\right)+F_{2}^{\left(1-\right)}\left(w,k\right)\geq r_{max}$.*

**Proof.**  Clearly, it follows that $F_{2}^{\left(1+\right)}\left(w,k\right)=0$, if and only if $wr_{new}\geq p_{in}\left(k\right)$. Simultaneously, since $\;p_{in}\left(k+1\right)\;$ and $r_{new}$ are integer multiples of $\frac{1}{M}$, we have $F_{2}^{\left(1-\right)}\left(w,k\right)=0\;\Leftrightarrow wr_{new}+\frac{1}{M}\leq p_{in}\left(k+1\right)\;\Leftrightarrow wr_{new}<p_{in}\left(k+1\right)\Leftrightarrow$, and no integer $k^{*}>k$ satisfies $p_{in}\left(k^{*}+1\right)\leq wr_{new}$.

If $F_{2}^{\left(1+\right)}\left(w,k\right)$ + $F_{2}^{\left(1-\right)}\left(w,k\right)\neq 0$, then it follows that either $F_{2}^{\left(1+\right)}\left(w,k\right)\neq 0$ or $F_{2}^{\left(1-\right)}\left(w,k\right)\neq 0$. Assuming $F_{2}^{\left(1+\right)}\left(w,k\right)\neq 0$, we derive the inequality $p_{in}\left(k\right)-wr_{new}>0$. Given that both $\;p_{in}\left(k\right)\;$ and $r_{new}$ are integer multiples of $\frac{1}{M}$, it can be concluded that $p_{in}\left(k\right)-wr_{new}\geq \frac{1}{M}$. Consequently, this leads to the result $Mr_{max}\left(p_{in}\left(k\right)-wr_{new}\right)\geq r_{max}$. Thus, we obtain $F_{2}^{\left(1+\right)}\left(w,k\right)+F_{2}^{\left(1-\right)}\left(w,k\right)\geq r_{max}$. If $F_{2}^{\left(1-\right)}\left(w,k\right)\neq 0$, we have $wr_{new}\geq p_{in}\left(k+1\right)$. This implies that $Mr_{max}\left(wr_{new}-p_{in}\left(k+1\right)\right)\geq 0$. Hence, we conclude that $\delta \left(Mr_{max}\left(wr_{new}-p_{in}\left(k+1\right)\right)+r_{max}\right)\geq r_{max}$. Therefore, the claim is proven. □

**Theorem** **9.** *For each $w,k\in \left[M\right]$ with w≥k, we have $F_{2}^{\left(2+\right)}\left(w,k\right)$ + $F_{2}^{\left(2-\right)}\left(w,k\right)=0$, if and only if $k=w^{\left(2\right)}$. Otherwise, it follows that $F_{2}^{\left(2+\right)}\left(w,k\right)+F_{2}^{\left(2-\right)}\left(w,k\right)\geq r_{max}$.*

**Proof.**  It is clear that $F_{2}^{\left(2+\right)}\left(w,k\right)=0$, if and only if $wr_{new}\geq p_{in}\left(k\right)+w_{in}$. Since $\;p_{in}\left(k+1\right)\;$ and $r_{new}$ are integer multiples of $\frac{1}{M}$, it follows that $F_{2}^{\left(2-\right)}\left(w,k\right)=0\;\Leftrightarrow wr_{new}+\frac{1}{M}p_{in}\left(k+1\right)+w_{in}\;\Leftrightarrow wr_{new}<p_{in}\left(k+1\right)+w_{in}\Leftrightarrow$, and no integer $k^{*}>k$ satisfies $p_{in}\left(k^{*}+1\right)+w_{in}\leq wr_{new}$

If $F_{2}^{\left(2+\right)}\left(w,k\right)$ + $F_{2}^{\left(2-\right)}\left(w,k\right)\neq 0$, it holds that either $F_{2}^{\left(2+\right)}\left(w,k\right)\neq 0$ or $F_{2}^{\left(2-\right)}\left(w,k\right)\neq 0$. Assuming $F_{2}^{\left(2+\right)}\left(w,k\right)\neq 0$, we have the inequality $\;wr_{new}<p_{in}\left(k\right)+w_{in}$. Since $r_{old}\;$, $r_{new}$ and $w_{in}$ are integer multiples of $\frac{1}{M}$, and we have $\;p_{in}\left(k\right)+w_{in}-wr_{new}\geq \frac{1}{M}$. Thus, we obtain $Mr_{max}\left(p_{in}\left(k\right)+w_{in}-wr_{new}\right)\geq r_{max}$ and $F_{2}^{\left(2+\right)}\left(w,k\right)+F_{2}^{\left(2-\right)}\left(w,k\right)\geq r_{max}$. If $F_{2}^{\left(2-\right)}\left(w,k\right)\neq 0$, we have $wr_{new}\geq p_{in}\left(k+1\right)+w_{in}$. This implies that $Mr_{max}\left(wr_{new}-p_{in}\left(k+1\right)-w_{in}\right)+r_{max}\geq r_{max}$. Hence, we conclude that $F_{2}^{\left(2+\right)}\left(w,k\right)+F_{2}^{\left(2-\right)}\left(w,k\right)\geq r_{max}$. □

### 3.3. The Third Hidden Layer of ASS-NN

In the third hidden layer, there are $M^{2}+M$ hidden neurons denoted as $F_{3}^{\left(1\right)}\left(w,k\right)$ for $w,k\in \left[M\right]$ with w≤k, and $F_{3}^{\left(2\right)}\left(w,k\right)$ for $w,k\in \left[M\right]$ with w>k. These neurons are defined as follows:

(24) $$\begin{array}{c} \begin{array}{c} F_{3}^{\left(1\right)}\left(w,k\right)=\delta \left(Mr_{max}-p_{in}\left(k\right)-M\left(F_{2}^{\left(1+\right)}\left(w,k\right)+F_{2}^{\left(1-\right)}\left(w,k\right)\right)\right)\\ h^{\left(1\right)}\left(w\right)=Mr_{max}-\sum_{k=w}^{M}F_{3}^{\left(1\right)}\left(w,k\right),\;w\in \left[M\right] \end{array}, \end{array}$$

(25) $$\begin{array}{c} \begin{array}{c} F_{3}^{\left(2\right)}\left(w,k\right)=\delta \left(k-M(F_{2}^{\left(2+\right)}\left(w,k\right)+F_{2}^{\left(2-\right)}\left(w,k\right))\right)\\ h^{\left(2\right)}\left(w\right)=p_{in}\left(\sum_{k=1}^{w}F_{3}^{\left(2\right)}\left(w,k\right)\right),\;w\in \left[M\right] \end{array}. \end{array}$$

The function of the third hidden layer is to calculate the values $p_{in}\left(w^{\left(1\right)}\right)$ and $p_{in}\left(w^{\left(2\right)}\right)$. The accuracy of this layer is guaranteed by the following two theorems.

**Theorem** **10.** *For each $w\in \left[M\right]$, if ${w\leq w}^{\left(1\right)}\leq M$, we have $h^{\left(1\right)}\left(w\right)=p_{in}\left(w^{\left(1\right)}\right)$. If $w^{\left(1\right)}>M$, it follows that $h^{\left(1\right)}\left(w\right)=Mr_{max}$.*

**Proof.**  If ${w\leq w}^{\left(1\right)}\leq M$, then by Theorem 8, we have $F_{3}^{\left(1\right)}\left(w,w^{\left(1\right)}\right)=Mr_{max}-p_{in}\left(w^{\left(1\right)}\right)$ and $F_{3}^{\left(1\right)}\left(w,k\right)=0$ for each $k\neq w^{\left(1\right)}$. Consequently, it follows that $h^{\left(1\right)}\left(w\right)=p_{in}\left(w^{\left(1\right)}\right)$. In the case where w(1)>M, it holds that $F_{2}^{\left(1+\right)}\left(w,k\right)+F_{2}^{\left(1-\right)}\left(w,k\right)\geq r_{max}$ for each *k*. This implies that $F_{3}^{\left(1\right)}\left(w,k\right)=0$. Thus, we conclude that $h^{\left(1\right)}\left(w\right)=Mr_{max}$. □

**Theorem** **11.** *For each $w\in \left[M\right]$, if $w^{\left(2\right)}\geq 1$, then $\sum_{k=1}^{w}F_{3}^{\left(2\right)}\left(w,k\right)=w^{\left(2\right)}$. If $w^{\left(2\right)}\leq 0$, then $\sum_{k=1}^{w}F_{3}^{\left(2\right)}\left(w,k\right)=0$.*

**Proof.**  If $w^{\left(2\right)}\geq 1$, then $\;F_{3}^{\left(2\right)}\left(w,w^{\left(2\right)}\right)=w^{\left(2\right)}$ and $\;F_{3}^{\left(2\right)}\left(w,k\right)=0$ for each $k\neq w^{\left(2\right)}$. Thus, we have $\sum_{k=1}^{w}F_{3}^{\left(2\right)}\left(w,k\right)=w^{\left(2\right)}$. If $w^{\left(2\right)}\leq 0$, then by Theorem 9, it follows that $\;F_{2}^{\left(2+\right)}\left(w,k\right)$ + $F_{2}^{\left(2-\right)}\left(w,k\right)\geq r_{\max}$ for each *k*. This implies $F_{3}^{\left(2\right)}\left(w,k\right)=0$. Thus, we obtain $\sum_{k=1}^{w}F_{3}^{\left(2\right)}\left(w,k\right)=0$. □

### 3.4. The Final Hidden Layer of ASS-NN

Obviously, the value $p_{in}\left(w^{\left(2\right)}\right)$ is equal to $h^{\left(2\right)}\left(w\right)$ according to Theorem 11. The function of the final hidden layer is to calculate the value of $max\{h^{\left(1\right)}\left(w\right),\delta \left(w_{in}+h^{\left(2\right)}\left(w\right)\right)\}$ and comprises *M* neurons, denoted as $F_{4}\left(w\right)$ for $w\in \left[M\right]$. Finally, we output the value of $p_{out}\left(w\right)$ for $w\in \left[M\right]$.

(26) $$\begin{array}{c} \begin{array}{c} F_{4}\left(w\right)=\delta \left(h^{\left(1\right)}\left(w\right)-\delta \left(w_{in}+h^{\left(2\right)}\left(w\right)\right)\right),\;w\in \left[M\right]\\ p_{out}\left(w\right)=F_{4}\left(w\right)+\delta \left(w_{in}+h^{\left(2\right)}\left(w\right)\right),\;w\in \left[M\right] \end{array}. \end{array}$$

**Theorem** **12.** *For each $w\in \left[M\right]$, the value $p_{out}\left(w\right)$ is equal to the value $max\{h^{\left(1\right)}\left(w\right),\delta \left(w_{in}+h^{\left(2\right)}\left(w\right)\right)\}$.*

**Proof.**  If $h^{\left(1\right)}\left(w\right)\geq \delta \left(w_{in}+h^{\left(2\right)}\left(w\right)\right)$, then $F_{4}\left(w\right)=\;h^{\left(1\right)}\left(w\right)-\delta \left(w_{in}+h^{\left(2\right)}\left(w\right)\right)$. Consequently, we have $p_{out}\left(w\right)=F_{4}\left(w\right)+\delta \left(w_{in}+h^{\left(2\right)}\left(w\right)\right)=\;h^{\left(1\right)}\left(w\right)$. Conversely, if $h^{\left(1\right)}\left(w\right)<\delta \left(w_{in}+h^{\left(2\right)}\left(w\right)\right)$, then $F_{4}\left(w\right)=0$. This leads to the conclusion that $p_{out}\left(w\right)=F_{4}\left(w\right)+\delta \left(w_{in}+h^{\left(2\right)}\left(w\right)\right)=\;\delta \left(w_{in}+h^{\left(2\right)}\left(w\right)\right)$. Thus, the value of $p_{out}\left(w\right)$ is equal to the value $max\{h^{\left(1\right)}\left(w\right),\delta \left(w_{in}+h^{\left(2\right)}\left(w\right)\right)\}$. □

Let $S=\{w_{1},\ldots ,w_{n}\}$ and *M* be fixed positive integers. Let $w^{OPT}$ represent the actual optimal solution to the SSP that does not exceed $wr_{n}$. The corresponding subset is denoted as $\;W^{OPT}$. Furthermore, define $\;W_{i}^{OPT}=W^{OPT}\cap \left[i\right]$, and $w_{i}^{OPT}=\sum_{j\in W_{i}^{OPT}}w_{j}$.

**Theorem** **13.** *For $i\in \left[n\right]$ and $w\leq \left\lceil \frac{w_{i}^{OPT}}{r_{i}}\right\rceil -1$, we have $p\left(w,i\right)\leq w_{i}^{OPT}$.*

**Proof.**  If $w\leq \left\lceil \frac{w_{i}^{OPT}}{r_{i}}\right\rceil -1$, we have

(27) $$\begin{array}{c} wr_{i}\leq \left(\left\lceil \frac{w_{i}^{OPT}}{r_{i}}\right\rceil -1\right)r_{i}=\left\lceil \frac{w_{i}^{OPT}}{r_{i}}\right\rceil r_{i}-r_{i}\leq w_{i}^{OPT} \end{array}.$$

Given that $p\left(w,i\right)\leq wr_{i}$, it follows that

(28) $$\begin{array}{c} p\left(w,i\right)\leq w_{i}^{OPT} \end{array}.$$

□

Let $w^{NN}$ be the approximate solution to ASS-NN and $w^{OPT}$ be the actual solution. We have the following theorem.

**Theorem** **14.** *The values of $w^{NN}$ and $w^{OPT}$ satisfy the following inequality: $w^{NN}\geq w^{OPT}\left(1-\varepsilon \right)$.*

**Proof.**  According to Theorem 13, it follows that $p\left(\left\lceil \frac{w^{OPT}}{r_{n}}\right\rceil -1,n\right)\leq w_{n}^{OPT}$, and we have the following inequalities:

(29) $$\begin{array}{c} w^{NN}\geq \left\lceil \frac{w^{OPT}}{r_{n}}-n-1\right\rceil r_{n}\geq w^{OPT}-\left(n+1\right)r_{n} \end{array}.$$

Given that $w^{OPT}\geq {\frac{w}{n}}^{*}$, $r_{n}={\frac{w}{M}}^{*}$, we have

(30) $$\begin{array}{c} \frac{w^{NN}}{w^{OPT}}\geq 1-\frac{n(n+1)}{M}\geq 1-\varepsilon \end{array}.$$

Consequently, we have $w^{NN}\geq w^{OPT}\left(1-\varepsilon \right)$. □

The depth of a single ASS-NN is six, comprising four hidden layers, one input layer, and one output layer. Consequently, the depth of the ASS-NN designed for solving SSPs is represented as 6n, where *n* denotes the number of set elements. Thus, the order of depth can be expressed as O(6n). The width of the ASS-NN is determined by the value of *M*. Given that the hidden layers with maximum width are identified as the second and third layers, we conclude that the width order for a single ASS-NN is $O\left(2{\left(M+2\right)}^{2}\right)$. Therefore, when unfolding the ASS-NN and treating it as a singular neural network executing an entire dynamic program, we derive a width complexity of $O\left(2n{\left(M+2\right)}^{2}\right)$.

## 4. Verification and Analysis with Examples

### 4.1. Example for SS-NN

To demonstrate that the SS-NN can effectively solve the SSP, we consider an illustrative example. Let $S=\left\{w_{1}=8,w_{2}=34,w_{3}=4,w_{4}=12,w_{5}=5,w_{6}=3\right\}$ and W=7. The solution processes of SS-NN is described as follows.

Iteration 1: The inputs of SS-NN are $s_{in}\left(-1,0\right),s_{in}\left(0,0\right),s_{in}\left(1,0\right),\ldots ,s_{in}\left(6,0\right),s_{in}\left(7,0\right)$ and $w_{1}$, respectively. The corresponding values are 0,1,0,…,0 and 8, respectively. By utilizing the first hidden layer of the SS-NN model, we determine that $F_{1}\left(-1,8\right)=F_{1}\left(0,8\right)=\cdots =F_{1}\left(6,8\right)=F_{1}\left(7,8\right)=-1$. Furthermore, it follows that $F_{4}\left(-1\right)=F_{4}\left(0\right)=F_{4}\left(1\right)=\;F_{4}\left(2\right)=\cdots =F_{4}\left(6\right)=F_{4}\left(7\right)=0$. Consequently, the outputs of SS-NN are $s_{out}\left(-1,1\right),s_{out}\left(0,1\right),s_{out}\left(1,1\right),\ldots ,s_{out}\left(6,1\right)$ and $s_{out}\left(7,1\right)$, respectively. The corresponding values for these outputs are as follows: 0,1,0,...,0 and 0, respectively.

Iteration 2: The inputs of SS-NN are 0,1,0,…,0 and 34. According to the SS-NN model, the outputs are $s_{out}\left(-1,2\right),s_{out}\left(0,2\right),s_{out}\left(1,2\right),\ldots ,s_{out}\left(6,2\right)$ and $s_{out}\left(7,2\right)$, respectively. The corresponding value for these outputs are 0,1,0,…,0 and 0, respectively.

Iteration 3: The inputs of SS-NN are 0,1,0,…,0 and 4. According to the SS-NN framework, the corresponding outputs are as follows: 0,1,0,0,0,1,0,0,0.

Iteration 4: The inputs for the SS-NN model are 0,1,0,0,0,1,0,0,0 and 12. Based on the SS-NN framework, the corresponding outputs are 0,1,0,0,0,1,0,0,0.

Iteration 5: The inputs for the SS-NN model are as follows: 0,1,0,0,0,1,0,0,0 and 5. According to the SS-NN model, the outputs are 0,1,0,0,0,1,1,0,0.

Iteration 6: The inputs of SS-NN are 0,1,0,0,0,1,1,0,0 and 3. Based on the SS-NN model, the outputs are 0,1,0,0,1,1,1,0,1.

The values $s_{out}\left(w,i\right)$ computed by the SS-NN are shown in Table 1. It is evident that the results obtained the SS-NN alignment with the actual outcomes. Notably, there exist subsets whose elements are equal to 3, 4, 5, and 7, respectively.

### 4.2. Example for ASS-NN

To demonstrate the capability of the ASS-NN in effectively solving the SSP, we consider an illustrative example. Let $S=\{w_{1}=7,w_{2}=34,w_{3}=4,w_{4}=12,w_{5}=5,w_{6}=3\}$, M=6. The solution processes of the ASS-NN is described as follows:

Iteration 1: The inputs of ASS-NN are $p_{in}\left(0\right),p_{in}\left(1\right),\ldots ,p_{in}\left(6\right)$ and $w_{in}$, respectively. The corresponding values are −∞,0,0,…,0 and 7, respectively. We have $r_{old}=0$ and $\;r_{new}=2$, as determined by the first hidden layer. Thus, we determine that $\;\;p_{out}\left(0\right)=-\infty ,p_{out}\left(1\right)=p_{out}\left(2\right)=p_{out}\left(3\right)=0$, and $p_{out}\left(4\right)=p_{out}\left(5\right)=p_{out}\left(6\right)=7$. To illustrate this process further, let us compute the value of $p_{out}\left(4\right)$. By analyzing the second hidden layer of ASS-NN, we obtain $w^{\left(1\right)}=6$ and $w^{\left(2\right)}=6$. Additionally, from the third hidden layer, we have $p_{in}\left(w^{\left(1\right)}\right)=p_{in}\left(w^{\left(2\right)}\right)=0$. It follows that $p_{in}\left(w^{\left(1\right)}\right)<\left(p_{in}\left(w^{\left(2\right)}\right)+w_{in}\right)$, and we conclude that the value of $p_{out}\left(4\right)$ is equal to 7.

Iteration 2: The inputs to the ASS-NN are −∞,0,0,0,7,7,7 and 34, respectively. We obtain $r_{old}=2$ and $r_{new}=7$ from the first hidden layer. Consequently, we obtain the following output values: $\;p_{out}\left(0\right)=-\infty$, $\;p_{out}\left(1\right)=p_{out}\left(2\right)=p_{out}\left(3\right)=p_{out}\left(4\right)=7$, $\;p_{out}\left(5\right)=34$, and $p_{out}\left(6\right)=41$. To illustrate the computing process, let us compute the value of $p_{out}\left(4\right)$. According to the second hidden layer of ASS-NN, we obtain $w^{\left(1\right)}=6$ and $w^{\left(2\right)}=0$. Furthermore, we have $p_{in}\left(w^{\left(1\right)}\right)=7$ and $p_{in}\left(w^{\left(2\right)}\right)=-\infty$ from the third hidden layer. This implies that $p_{in}\left(w^{\left(1\right)}\right)>\left(p_{in}\left(w^{\left(2\right)}\right)+w_{in}\right)$. It follows that $p_{out}\left(4\right)$ = 7.

Iteration 3: The inputs of ASS-NN are −∞,7,7,7,7,34,41 and 4, respectively. Consequently, we obtain the following output values: $r_{old}=7$ and $r_{new}=8$ from the first hidden layer. Thus, we have $p_{out}\left(0\right)=-\infty$, $\;p_{out}\left(1\right)=7,p_{out}\left(2\right)=p_{out}\left(3\right)=p_{out}\left(4\right)=11$, $p_{out}\left(5\right)=38$, and $p_{out}\left(6\right)=45$. Let us consider how to compute the value of $p_{out}\left(4\right)$. By analyzing the second hidden layer of ASS-NN, we determine that $w^{\left(1\right)}=4$ and $w^{\left(2\right)}=4$. Furthermore, we have $p_{in}\left(w^{\left(1\right)}\right)=7$ and $p_{in}\left(w^{\left(2\right)}\right)=7$ from the third hidden layer. Given that $p_{in}\left(w^{\left(1\right)}\right)<\left(p_{in}\left(w^{\left(2\right)}\right)+w_{in}\right)$, we conclude that the value $p_{out}\left(4\right)$ is equal to 11.

Iteration 4: The inputs of ASS-NN are −∞,7,11,11,11,38,45 and 12, respectively. Utilizing the first hidden layer, we obtain $r_{old}=8$ and $r_{new}=10$. Consequently, we have the following output values: $p_{out}\left(0\right)=-\infty$, $p_{out}\left(1\right)=7$, $p_{out}\left(2\right)=19$, $p_{out}\left(3\right)=23$, $p_{out}\left(4\right)=38$, $p_{out}\left(5\right)=50$, and $p_{out}\left(6\right)=57$. To illustrate computing process, let us compute the value of $p_{out}\left(4\right)$. We can derive $w^{\left(1\right)}=5$ and $w^{\left(2\right)}=3$ from the second hidden layer of ASS-NN. Furthermore, we conclude that $p_{in}\left(w^{\left(1\right)}\right)=38$ and $p_{in}\left(w^{\left(2\right)}\right)=11$ from the third hidden layer. Since $p_{in}\left(w^{\left(1\right)}\right)>\left(p_{in}\left(w^{\left(2\right)}\right)+w_{in}\right)$, we can obtain the value $p_{out}\left(4\right)$, which is equal to 38.

Iteration 5: The inputs of ASS-NN are −∞,7,19,23,38,50,57 and 5, respectively. We have $r_{old}=10$ and $r_{new}=11$ as determined by the first hidden layer. Consequently, we obtain the following output values: $p_{out}\left(0\right)=-\infty$, $p_{out}\left(1\right)=7$, $p_{out}\left(2\right)=19$, $p_{out}\left(3\right)=28$, $p_{out}\left(4\right)=43$, $p_{out}\left(5\right)=55$, and $p_{out}\left(6\right)=62$. To further illustrate this process, let us compute the value of $p_{out}\left(4\right)$. According to the second hidden layer of ASS-NN, we obtain $w^{\left(1\right)}=4$ and $w^{\left(2\right)}=4$. Additionally, from the third hidden layer we determine that $p_{in}\left(w^{\left(1\right)}\right)=38$ and $p_{in}\left(w^{\left(2\right)}\right)=38$. Given that $p_{in}\left(w^{\left(1\right)}\right)<\left(p_{in}\left(w^{\left(2\right)}\right)+w_{in}\right)$, it follows that the value $p_{out}\left(4\right)$ is equal to 43.

Iteration 6: The inputs of ASS-NN are −∞,7,19,28,43,55,62 and 3, respectively. Utilizing the first hidden layer, we obtain $r_{old}=11$ and $r_{new}=11$. Consequently, we have the following output values: $p_{out}\left(0\right)=-\infty$, $p_{out}\left(1\right)=10$, $p_{out}\left(2\right)=22$, $p_{out}\left(3\right)=31$, $p_{out}\left(4\right)=43$, $p_{out}\left(5\right)=55$, and $p_{out}\left(6\right)=65$. Take $p_{out}\left(4\right)$ as an example. By analyzing the second hidden layer of ASS-NN, we determine that $w^{\left(1\right)}=4$ and $w^{\left(2\right)}=3$. Additionally, from the third hidden layer, we have that $p_{in}\left(w^{\left(1\right)}\right)=43$ and $p_{in}\left(w^{\left(2\right)}\right)=28$. Given that $p_{in}\left(w^{\left(1\right)}\right)>\left(p_{in}\left(w^{\left(2\right)}\right)+w_{in}\right)$, we can obtain the value $p_{out}\left(4\right)$, which is equal to 43.

The values $p_{out}\left(w,i\right)$ computed by the ASS-NN are shown in Table 2. Given a positive integer *P*, we can identify a subset of *S* such that the sum of its elements is closest to *P* without exceeding *P*. For instance, if we aim to determine a subset of *S*, the sum of the subset elements is closest to 33 but no more than 33. Firstly, we observe the value closest to 33 but no more than 33 in Table 2 is 31, and the corresponding function is $p_{out}\left(3,6\right)$. The solution process for identifying the subset associated with $p_{out}\left(3,6\right)$ proceeds as follows:

1. Given that $p_{out}\left(3,6\right)=\max\left\{p_{out}\left(3,5\right),\delta \left(p_{out}\left(3,5\right)+3\right)\right\}$$=p_{out}\left(3,5\right)+3$, it follows that 3 belongs to the required subset.
2. Given that $p_{out}\left(3,5\right)=\max\left\{p_{out}\left(3,4\right),\delta \left(p_{out}\left(3,4\right)+5\right)\right\}$$=p_{out}\left(3,4\right)+5$, it follows that 5 belongs to the required subset.
3. Given that $p_{out}\left(3,4\right)=\max\left\{p_{out}\left(4,3\right),\delta \left(p_{out}\left(4,3\right)+12\right)\right\}$$=p_{out}\left(4,3\right)+12$, 12 also belongs to our desired subset.
4. Given that $p_{out}\left(4,3\right)=\max\left\{p_{out}\left(4,2\right),\delta \left(p_{out}\left(4,2\right)+4\right)\right\}$$=p_{out}\left(4,2\right)+4$, 4 belongs to the required subset.
5. Given that $p_{out}\left(4,2\right)=\max\left\{p_{out}\left(6,1\right),\delta \left(p_{out}\left(0,1\right)+34\right)\right\}$$=p_{out}\left(6,1\right)$, 34 does not belong in our required set.
6. Given that $p_{out}\left(6,1\right)=\max\left\{p_{out}\left(6,0\right),\delta \left(p_{out}\left(6,0\right)+7\right)\right\}$$=p_{out}\left(6,0\right)+7$, we conclude that 7 is included in the required subset.
7. Given that the second argument of $p_{out}\left(6,0\right)$ is zero, the solution process concludes here. Ultimately, we determine that the required subset of is $\left\{3,5,12,4,7\right\}$.

Since the value $p_{out}\left(w,i\right)$ of ASS-NN serves as an approximation to the SSP, there may exist a discrepancy between $p_{out}\left(w,i\right)$ and the actual optimal solution. The values $p_{out}\left(2,6\right)$, $p_{out}\left(3,6\right)$, $p_{out}\left(5,6\right)$, and $p_{out}\left(6,6\right)$ are equal to the actual optimal solution, and the error ratio is zero. The errors between $p_{out}\left(1,6\right)$, $p_{out}\left(4,6\right)$, and the actual optimization are 1. Specifically, the error ratio of $p_{out}\left(1,6\right)$ is 0.091, and $p_{out}\left(4,6\right)$ is 0.023.

### 4.3. Error Analysis

In order to assess the effectiveness of the proposed ASS-NN in addressing subset problems, we evaluate the degree of error between the approximate solution generated by ASS-NN and the actual optimal solution. Let $w^{NN}$ be the approximation solution with ASS-NN and $w^{OPT}$ be the actual optimal solution. The degree of error δ is calculated using Equation (31).

(31) $$\begin{array}{c} \delta =\frac{w^{OPT}-w^{NN}}{w^{OPT}}. \end{array}$$

**Figure 7:**
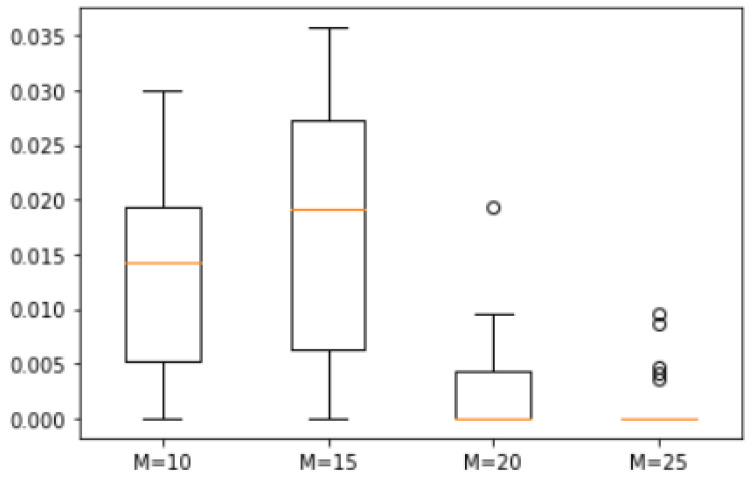
Error of Example 1.

**Example** **1.** *A set of 20 natural numbers was randomly generated within a range from 1 to 50, resulting in the set S = {50, 48, 46, 45, 44, 43, 32, 30, 29, 25, 24, 20, 16, 15, 14, 13, 10, 6, 4, 1}. We analyzed the error degree across four scenarios with M values of 10, 15, 20, and 25. The corresponding box plots are shown in Figure 7. The maximum errors between the approximate solutions derived from ASS-NN and the actual optimal solution were found to be 3%, 3.5%, 2%, and 1%, respectively. In the scenario where M equals 25, only 5 out of 25 approximate solutions deviated from the actual optimal solution. The median difference across all four cases remained within a margin of 2%.*

**Figure 8:**
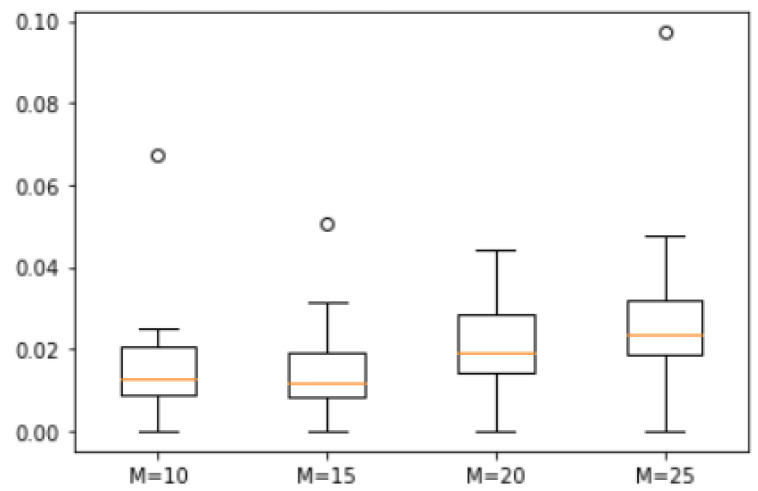
Error of Example 2.

**Example** **2.** *A total of 20 natural numbers were randomly generated within a range from 1 to 100 to form the set S = {90, 72, 65, 61, 59, 58, 57, 56, 47, 44, 43, 38, 37, 34, 30, 29, 22, 20, 13, 6}. We analyzed the error degrees for four different values of M = 10, 15, 20 and 25, and the corresponding box plot is shown in Figure 8. The maximum errors observed between the approximate solutions derived from ASS-NN and those representing actual optimal solutions are recorded as follows: 6.7%, 5%, 4.5%, and 10%, respectively. These findings indicate that while there exists a minimal median difference across all four scenarios examined here, the variability in error tends to escalate with increasing values of M.*

**Figure 9:**
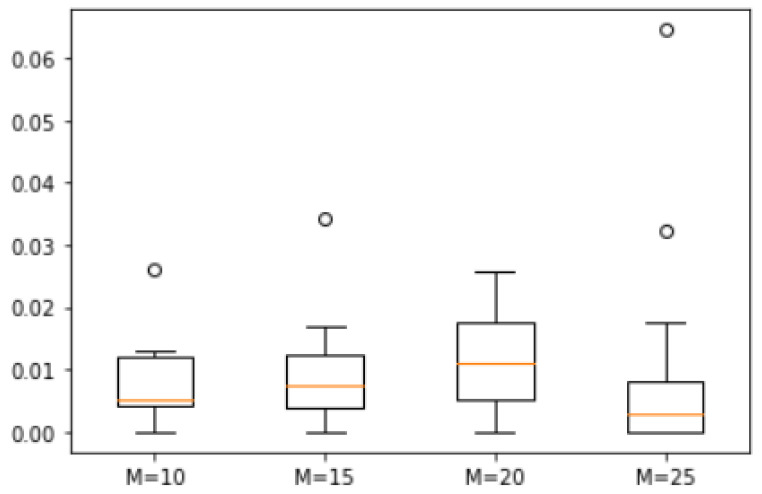
Error of Example 3.

**Example** **3.** *We randomly generated 25 natural numbers between 1 and 50, resulting in the set S = {50, 49, 48, 47, 46, 45, 43, 42, 40, 39, 36, 35, 33, 32, 30, 26, 22, 21, 20, 18, 14, 13, 10, 8, 3}. We analyzed the error degree across four cases with M = 10, 15, 20, and 25. The corresponding box plot is illustrated in Figure 9. The maximum errors between the approximate solutions obtained through ASS-NN and the actual optimal solution were found to be 2.7%, 3.5%, 2.7%, and 6.8%, respectively. Among these four cases of analysis for different values of M, it was observed that the median for M = 25 yielded the lowest value. Furthermore, the median differences across all four scenarios remained within a margin of less than or equal to 1%.*

**Figure 10:**
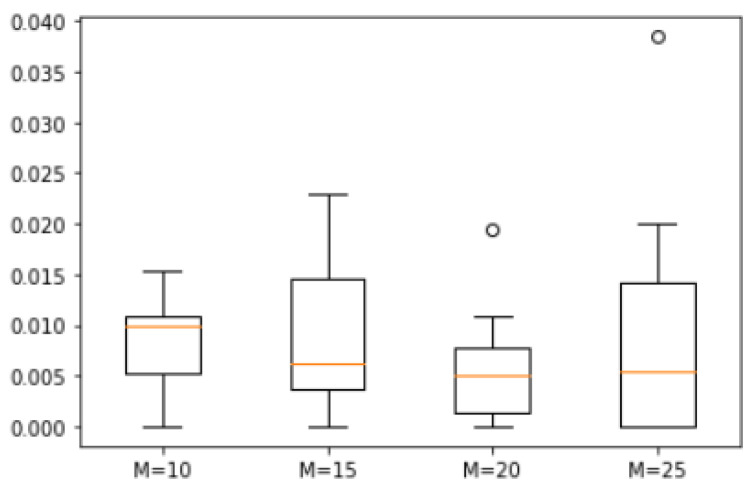
Error of Example 4.

**Example** **4.** *A set of 25 natural numbers was randomly generated within a range from 1 to 100, resulting in the set S = {100, 95, 93, 91, 82, 81, 79, 71, 70, 67, 65, 54, 50, 46, 44, 41, 38, 31, 27, 25, 19, 11, 10, 4, 2}. We analyzed the error degree across four scenarios with M values of 10, 15, 20, and 25. The corresponding box plot is illustrated in Figure 10. The maximum errors between the approximate solutions derived from ASS-NN and the actual optimal solution are recorded as 1.5%, 2.3%, 2%, and 3.8%, respectively. Among these cases examined, M = 15 yielded the lowest median value. Furthermore, the median differences across all four scenarios remained within a margin of 0.5%.*

**Figure 11:**
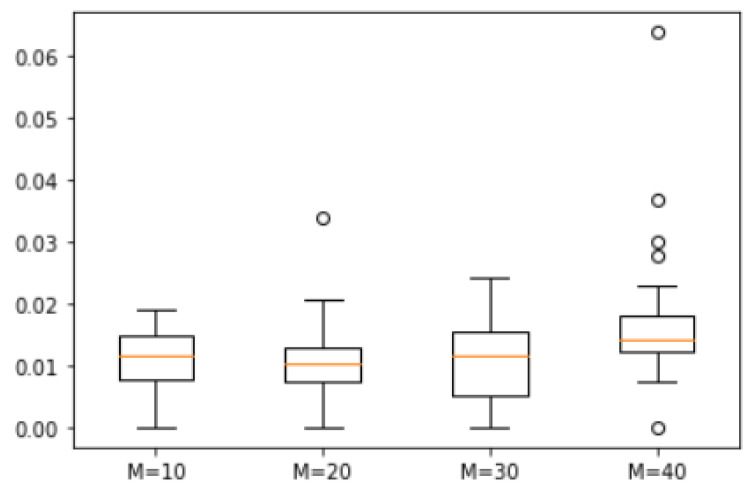
Error of Example 5.

**Example** **5.** *We randomly generated 25 natural numbers between 1 and 200, resulting in the set S = {196, 194, 193, 177, 172, 165, 157, 155, 152, 133, 130, 126, 108, 101, 98, 91, 76, 60, 57, 52, 50, 27, 9, 4, 1}. We analyzed the error degree across four cases with M = 10, 20, 30, and 40. The corresponding box plots are illustrated in Figure 11. The maximum errors between the approximate solutions obtained by ASS-NN and the actual optimal solution were found to be 1.9%, 3.5%, 2.5%, and 6.8%, respectively. Among these four cases, a median for M = 20 was observed to be the lowest. Furthermore, the median differences among all four cases remained within a range of 0.5%.*

**Figure 12:**
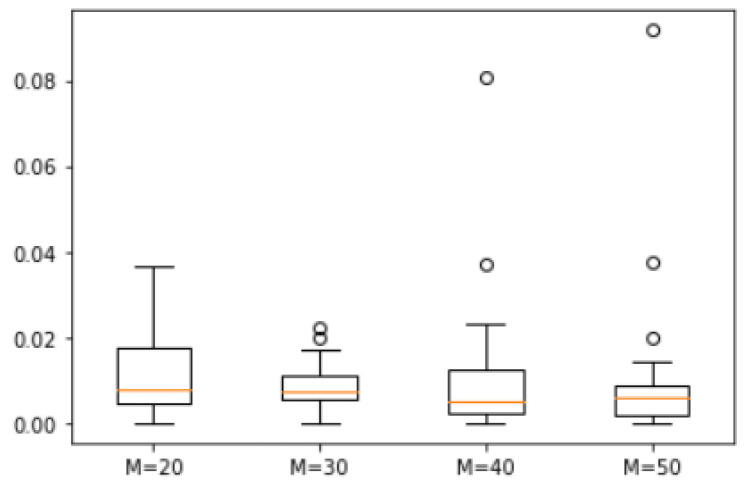
Error of Example 6.

**Example** **6.** *We randomly generated 25 natural numbers between 1 and 500, resulting in the set S = {461, 450, 409, 391, 362, 349, 310, 293, 291, 276, 263, 238, 202, 199, 135, 128, 121, 120, 119, 114, 54, 48, 43, 37, 9}. We analyzed the error degree across four scenarios with M = 20, 30, 40, and 50. The corresponding box plots are shown in Figure 12. The maximum errors between the approximate solutions obtained through ASS-NN and the actual optimal solution were found to be 3.8%, 2.6%, 8%, and 9.2%, respectively. Among these cases, a median of M = 40 was observed to be the lowest. Furthermore, the median differences across all four scenarios remained within a range of 0.5%.*

According to the box diagram, we observed that an increase in the *M* value corresponds to a greater number of abnormal values. However, the average difference in error degree for the same dataset across different *M* values remains minimal. Furthermore, it is evident that the discrepancy between the approximate solution and the actual solution aligns with the theoretical error degree.

Consider Example 6 again, where *M* = 50. In this scenario, the approximate solutions generated by ASS-NN alongside the actual optimal solution are presented in Table 3. Among the total of fifty solutions evaluated by ASS-NN methods; five of these correspond exactly with the actual optimal solution. Furthermore, nineteen solutions exhibited an error value ranging from 1 to 10, twelve solutions had an error value from 11 to 20, and fourteen solutions presented an error value exceeding 20. The maximum observed error value was 46, occurring at *w* = 44, while the highest degree of error reached approximately 9.17% when *w* = 2. These results indicate that the approximate solution method based on ASS-NN effectively addresses SSPs while maintaining a minimal discrepancy between approximate and actual optimal solutions.

## 5. Discussion

The SSP is a classical combinatorial optimization issue. It is not merely an abstract computational conundrum but also a juncture where theory and practice converge. Its research consistently promotes algorithmic innovation and offers methodological support for practical engineering problems. As of now, the classical approaches for solving the SSP encompass dynamic programming, genetic algorithms, quantum algorithms, double list algorithms, and others. The merit of the dynamic programming method lies in constructing solutions based on recurrence relations and being capable of attaining the optimal solution. Nevertheless, its time and space complexities escalate rapidly with the scale, thus being suitable for small-scale problems [16]. Quantum algorithms can concurrently explore multiple candidate solutions via superposition states and effectively search for the optimal solution in the solution space. However, they are vulnerable to noise interference, have high implementation complexity, and rely on dedicated quantum devices [26]. Genetic algorithms exhibit strong adaptability to large-scale problems but are sensitive to parameters and prone to getting trapped in local optima [15]. The double list algorithm can significantly reduce time complexity but has a high space complexity [5]. Simultaneously, all these methods encounter the combinatorial explosion problem resulting from traversing all possible combination spaces and a lack of generalization ability for similar problems.

Based on the powerful pattern learning capability and end-to-end mapping solution approach of neural network models, and given that RNN can simulate the process of constructing solutions based on recurrence relations in dynamic programming [17], this paper theoretically constructs an RNN for solving the SSP and proves its correctness. For the problem of whether there exists a subset in a set such that the sum of all elements in the subset equals a specified *w* value, recent research by [24] from the perspective of geometry, which interpreted SSP as a problem of deciding whether the intersection of the positive unit hypercube with the hyperplane contains at least a vertex, solved the problem precisely. The SS-NN network we constructed can solve the problem precisely, too. In Example 1 of Section 4, we present the solution process of SS-NN. The results indicate that SS-NN can not only provide the answer as to whether a subset exists whose sum of all elements equals a specified *w* value but also yield solution results for all problems with values less than *w*. However, although both methods can yield exact solutions, the method in the literature [24] can be applied to the simultaneous SSP.

For the problem of finding a subset such that the sum of elements in the subset is closest to but does not exceed a specified value, considering that constructing a neural network model to solve the exact value would be highly complex and demand a considerable amount of computational resources, this paper constructs the ASS-NN model for solving the approximate solution of the sub-problem. Compared to the model for solving the exact solution, this model is simpler but can determine an approximate solution that is very close to the optimal solution. For instance, in Table 3, $w^{NN}$ represents the approximate solution obtained by the ASS-NN model, while $w^{OPT}$ represents the actual optimal solution of the problem. In some cases, the solution obtained by the ASS-NN model is the optimal solution. Experimental results show that the maximum error rate between the approximate solution obtained by the ASS-NN model and the optimal solution is approximately 9.17%. Once it is theoretically ensured that the constructed neural network model can solve the problem, then in practice, by training the model and utilizing the end-to-end mapping approach of the model, it is possible to bypass explicitly traversing all possible combination spaces, thereby avoiding the combinatorial explosion. This is also the distinction between solving the SSP through the RNN model and traditional heuristic algorithms [11] and dynamic programming [19] and represents another approach to solving large-scale SSPs. However, the accuracy of the solution obtained by ASS-NN still needs to be improved.

## 6. Conclusions

In light of the deficiencies of existing dynamic programming, genetic algorithms, and quantum algorithms when addressing the SSP, and inspired by the work of C. Hertrich et al. [37], we proposed two models, namely SS-NN and ASS-NN, for simulating dynamic programming to solve the SSP. Stringent mathematical derivations demonstrate that the proposed neural network models can precisely solve the SSP. Among them, the SS-NN model can determine whether there exists a subset within a set such that the sum of all elements in this subset equals a specified value, while the ASS-NN model is capable of providing an approximate solution that is extremely close to the optimal solution for the problem of finding a subset whose sum of elements is closest to but does not exceed the specified value. Experimental results indicate that the errors between the approximate solutions obtained by the ASS-NN model and the actual optimal solutions are relatively small and highly consistent with theoretical expectations. Our research reveals that RNNs provide a novel approach to resolving the SSP. This approach not only constructs the solution process by emulating the recursive relationship in dynamic programming but also utilizes neural network models to learn the latent patterns in the problem and circumvents the combinatorial explosion problem caused by explicitly traversing all possible combination spaces through an end-to-end mapping approach. However, in practical applications, using RNNs to solve the SSP still confronts several challenges, mainly the following: (1) due to strong data dependence, the cost of generating high-quality training samples is relatively high; (2) for certain SSPs, if an exact solution is sought, it is necessary to construct a complex network model, thereby triggering the demand for a substantial amount of computing resources; (3) the accuracy of the approximate solutions still awaits further enhancement. In future research, we will introduce adversarial generative networks [39] to generate high-quality training samples and integrate the attention mechanism [40] to improve the solution accuracy of the model.

## Figures and Tables

**Table 1:** The value of $s_{out}\left(w,i\right)$.

	*w*	−1	0	1	2	3	4	5	6	7
*i*										
**0**		0	1	0	0	0	0	0	0	0
**1**		0	1	0	0	0	0	0	0	0
**2**		0	1	0	0	0	0	0	0	0
**3**		0	1	0	0	0	0	0	0	0
**4**		0	1	0	0	0	1	0	0	0
**5**		0	1	0	0	0	1	1	0	0
**6**		0	1	0	0	1	1	1	0	1

**Table 2:** The value of $p_{out}\left(w,i\right)$.

	*w*	0	1	2	3	4	5	6
*i*								
**0**		−∞	0	0	0	0	0	0
**1**		−∞	0	0	0	7	7	7
**2**		−∞	7	7	7	7	34	41
**3**		−∞	7	11	11	11	38	45
**4**		−∞	7	19	23	38	50	57
**5**		−∞	7	19	28	43	55	62
**6**		−∞	10	22	31	43	55	65

**Table 3:** Results of Example 6 with M = 50.

*w*	1	2	3	4	5	6	7	8	9	10	11	12
$w^{NN}$	102	198	326	436	540	654	757	862	971	1090	1175	1289
$w^{OPT}$	106	218	327	436	545	654	763	872	981	1090	1199	1304
err	4	20	1	0	5	0	6	10	10	0	24	15
*w*	**13**	**14**	**15**	**16**	**17**	**18**	**19**	**20**	**21**	**22**	**23**	**24**
$w^{NN}$	1417	1525	1614	1725	1846	1961	2053	2166	2274	2383	2499	2593
$w^{OPT}$	1417	1526	1635	1744	1853	1962	2071	2180	2289	2398	2507	2616
err	0	1	21	19	7	1	18	14	15	15	8	23
*w*	**25**	**26**	**27**	**28**	**29**	**30**	**31**	**32**	**33**	**34**	**35**	**36**
$w^{NN}$	2708	2828	2942	3044	3158	3263	3365	3483	3569	3683	3797	3898
$w^{OPT}$	2725	2834	2943	3052	3161	3270	3379	3488	3597	3706	3815	3924
err	17	6	1	8	3	7	14	5	28	23	18	26
*w*	**37**	**38**	**39**	**40**	**41**	**42**	**43**	**44**	**45**	**46**	**47**	**48**
$w^{NN}$	4007	4111	4208	4355	4432	4546	4685	4750	4870	4990	5109	5223
$w^{OPT}$	4033	4142	4251	4360	4469	4578	4687	4796	4905	5014	5123	5231
err	26	31	43	5	37	32	2	46	35	24	14	8
*w*	**49**	**50**										
$w^{NN}$	5308	5422										
$w^{OPT}$	5377	5422										
err	29	0										

## Data Availability

The data that support the findings of this study are available from the corresponding author upon request. There are no restrictions on data availability.
